# Ultrasound Diagnosis and Near-Infrared Spectroscopy in the Study of Encephalopathy in Neonates Born under Asphyxia: Narrative Review

**DOI:** 10.3390/children11050591

**Published:** 2024-05-14

**Authors:** Simeon N. Lavrentev, Anastasia S. Petrova, Olga F. Serova, Polina Vishnyakova, Maxim V. Kondratev, Anastasia S. Gryzunova, Nina I. Zakharova, Victor V. Zubkov, Denis N. Silachev

**Affiliations:** 1 The State Budgetary Institution, Moscow Regional Perinatal Center, 143912 Balashikha, Russia; semyonlavrentev@mail.ru (S.N.L.); trifonovaas@gmail.com (A.S.P.); olga-serova@yandex.ru (O.F.S.); mama-x@yandex.ru (M.V.K.); nkvaselisa@gmail.com (A.S.G.); 2 Research Clinical Institute of Childhood of the Moscow Region, 115093 Moscow, Russia; oor@bk.ru (N.I.Z.); v_zubkov@oparina4.ru (V.V.Z.); 3 V.I. Kulakov National Medical Research Center for Obstetrics Gynecology and Perinatology, Ministry of Healthcare of the Russian Federation, 117997 Moscow, Russia; p_vishnyakova@oparina4.ru; 4 Research Institute of Molecular and Cellular Medicine, Peoples’ Friendship University of Russia (RUDN University), 117198 Moscow, Russia; 5 A.N. Belozersky Institute of Physico-Chemical Biology, Lomonosov Moscow State University, 119991 Moscow, Russia

**Keywords:** hypoxic-ischemic encephalopathy, neonatal encephalopathy, brain ultrasound, NIRS, cerebral perfusion, newborns

## Abstract

Brain injury resulting from adverse events during pregnancy and delivery is the leading cause of neonatal morbidity and disability. Surviving neonates often suffer long-term motor, sensory, and cognitive impairments. Birth asphyxia is among the most common causes of neonatal encephalopathy. The integration of ultrasound, including Doppler ultrasound, and near-infrared spectroscopy (NIRS) offers a promising approach to understanding the pathology and diagnosis of encephalopathy in this special patient population. Ultrasound diagnosis can be very helpful for the assessment of structural abnormalities associated with neonatal encephalopathy such as alterations in brain structures (intraventricular hemorrhage, infarcts, hydrocephalus, white matter injury) and evaluation of morphologic changes. Doppler sonography is the most valuable method as it provides information about blood flow patterns and outcome prediction. NIRS provides valuable insight into the functional aspects of brain activity by measuring tissue oxygenation and blood flow. The combination of ultrasonography and NIRS may produce complementary information on structural and functional aspects of the brain. This review summarizes the current state of research, discusses advantages and limitations, and explores future directions to improve applicability and efficacy.

## 1. Introduction

### 1.1. Neonatal Encephalopathy

Neonatal encephalopathy (NE) is a clinical syndrome of neurological dysfunction in newborns, characterized by various signs depending on their severity. In medical practice, the term “neonatal encephalopathy” is used to describe the neurological condition of a newborn, regardless of its underlying cause [1]. The estimated frequency of NE is 2 to 9 cases per 1000 live births [2]. NE can result from acute or chronic hypoxic-ischemic injury, developmental brain defects, vascular damage (including stroke), congenital metabolic disorders, and other causes [3,4]. One of the most common causes of neonatal encephalopathy in newborns is birth asphyxia. In this case, the clinical diagnosis of asphyxia is not an isolated central nervous system lesion, but a chain of interconnected events from adaptation to decompensation involving all organs and systems of the fetus and newborn [5]. Adaptation initiates with activation of the sympathetic-adrenal system by adrenal hormones and cytokines, as well as an increase in circulating red blood cells, heart rate, and possibly systolic pressure. Prolonged hypoxia promotes the involvement of an energetically unfavorable pathway of carbohydrate metabolism—anaerobic glycolysis. The cardiovascular response is to redirect blood flow so that vital organs such as the brain, heart, adrenal glands, and diaphragm are prioritized and the oxygen supply to the skin, lungs, gut, muscles, kidneys, and other tissues is reduced. Failure of these adaptive mechanisms can lead to destabilization, as the newborn compensatory abilities are limited. This leads to exacerbated metabolic acidosis, which promotes the activation of plasma proteases and proinflammatory factors, damages cell membranes, and triggers dyselectrolythemia. These effects exacerbate damage to critical systems such as the central nervous system, cardiovascular system, kidneys, and adrenal glands, potentially leading to multiple organ failure.

### 1.2. Hypoxic-Ischemic Encephalopathy as an Example of Neonatal Encephalopathy

The term “birth asphyxia”, which refers to impaired placental perfusion and gas exchange leading to hypoxia, ischemia, and acidosis, has historically been used to describe hypoxic-ischemic encephalopathy (HIE) [6]. However, the use of the term HIE is recommended when there are clear signs of antenatal hypoxia or intranatal asphyxia as the primary cause of NE [7,8]. The role of the placenta in normal fetal brain development is just beginning to be established and is the focus of a new field known as neuroplacentology [9]. A number of studies show a connection between placental pathology and HIE development [10,11,12]. According to published data from a cohort study at a single center involving 90 babies, 5% to 20% of neonatal HIE cases are attributed to hypoxic-ischemic injury in the antenatal period, while 56% are associated with hypoxic-ischemic events in the intranatal period. However, it is important to note that these statistics may not reflect the full challenge in determining the timing of hypoxic-ischemic brain injuries, given the limited scope of the study [13]. In economically developed countries, the prevalence of HIE in newborns is 1 per 1000 live births. In contrast, in economically underdeveloped countries, this rate ranges from 5 to 40 cases per 1000 live births [14]. Moreover, up to 60% of newborns who experience severe HIE die or develop profound disability [15]. Among those who experience severe HIE, 27% of children die by the age of 3 years old [16], and one-third of newborns with HIE, despite treatment, develop neurological symptoms of varying severity [17]. HIE severity is clinically evaluated using the Sarnat grading system, which is divided into grades I (mild), II (moderate), and III (severe) depending on HIE`s manifestation and duration [18].

In the absence of treatment, 53% to 66% of patients with moderate to severe HIE die or develop severe neurological symptoms [19]. The implementation of therapeutic hypothermia in neonates with moderate to severe hypoxic-ischemic encephalopathy has been demonstrated to reduce the severity of neurological disorders. However, its efficacy in reducing mortality in neonates, infants, and children is not consistently observed across all cases. The level of staff training and experience also affects the effectiveness of the therapy [20]. Among the causes associated with neonatal HIE, several obvious and less obvious factors should be highlighted. First and foremost, attention should be paid to the course of pregnancy and childbirth history. According to various authors, HIE is more likely to develop in newborns whose mothers were not registered with the women’s consultation or rarely visited a gynecologist during pregnancy, as well as those with a history of diabetes and gestational hypertension. Additionally, the age of mothers of newborns who later develop HIE is over 35, and this category of mothers has a complicated obstetric history [21]. Furthermore, another significant cause of neonatal HIE is the presence of maternal infectious diseases. According to the meta-analysis by Thayyil et al., in low-income and middle-income countries the implementation of therapeutic hypothermia in newborns with HIE not only does not improve neurological outcomes in this patient population but is associated with an increased risk of death and unfavorable outcomes, and more severe HIE is associated with the course and realization of intrauterine infection [22].

## 2. Features of Neonatal Hemodynamics

### 2.1. Cerebral Blood Flow in Normal Conditions and Hypoxic-Ischemic Injury

There is a direct relationship between the degree of brain injury during hypoxia-ischemia and the development of circulatory insufficiency. In general, neonatal hemodynamics undergo tremendous changes after a physiologically normal pregnancy, at the time of delivery, and in the first hours after birth. The first breath and an increase in the partial pressure of oxygen in the blood vessels, clamping of the umbilical cord, and activation of pulmonary blood flow with the exclusion of the placenta from blood flow result in a decrease in pulmonary and peripheral vascular resistance. At birth, the direction of blood flow through the arterial duct changes. Initially, it flows from right to left, then becomes bidirectional, and eventually transitions to a left-to-right flow, which leads to its closure within the first day of life. In the first 20 min after birth, heart rate decreases and ventricular stroke volume increases in healthy newborns. Mean blood flow velocity in the middle cerebral artery (MCA) decreases (from 34 ± 13 to 24 ± 7 cm/s) in inverse proportion to blood flow through the patent ductus arteriosus (PDA). With the closure of the PDA, left ventricular output increases along with an increase in systemic arterial pressure [23,24,25]. These physiologic changes may be influenced by several adverse factors, including premature detachment of a normally located placenta, preterm delivery, duration of umbilical cord compression, preeclampsia, chorioamnionitis, birth asphyxia, congenital pneumonia, and early neonatal sepsis [26]. 

The blood supply to brain cells is provided by a system of vessels controlled by a complicated regulatory system to ensure stable perfusion. Cerebral blood flow is controlled by numerous internal and external factors, such as intravascular and intracranial pressure, hematocrit level, and blood gases, such as CO_2_ and oxygen. A study of the characteristics of cerebral hemodynamics changes in uncomplicated births is necessary for adequate assessment of perfusion changes due to pre-, peri-, and postpartum brain injury [27]. One of the most important mechanisms of cerebral perfusion is autoregulation. Cerebral autoregulation describes the brain’s ability to maintain constant blood flow despite physiological fluctuations in arterial pressure, such as changes in position, tension, or stress. Impaired cerebral pressure autoregulation leads to secondary brain damage in children with HIE [28]. After asphyxia, therapeutic intervention may include adjustment of hemodynamic parameters to improve autoregulatory function. Depending on the clinical situation, either vasoactive drugs or intravascular volume support can be used [29]. Blood pressure deviations below MAPOPT (mean arterial pressure optimized for autoregulation) were associated with greater white matter and paracentral gyri damage in children with HIE. Maintaining blood pressure within the MAPOPT is associated with less damage to the white matter, putamen, globus pallidus, and brainstem [30].

Spectral coherence measurements between MAP (mean arterial pressure) and NIRS HbD (cerebral oxyhemoglobin–deoxyhemoglobin measured by near-infrared spectroscopy), which indicate impaired cerebral autoregulation, show differences in newborns diagnosed with HIE. There are differences between those who either died or suffered moderate to severe traumatic brain injury as identified by MRI and those who survived with mild to moderate injury. In particular, the infants with unfavorable outcomes had elevated pressure-passive index, suggesting a prolonged and more intense state of cerebral pressure passivity following hypoxia-ischemia [31]. In an observational pilot study of neonates treated with hypothermia for HIE, researchers found an association between the regulation of blood pressure relative to optimal MAP and the extent of brain injury. Brain injury was assessed using the Neonatal Research Network (NRN) global score and regional mean diffusivity (MD) scalars. A longer duration of maintaining blood pressure within the optimal MAP range correlated with reduced NRN brain injury scores and increased MD in the left and the right anterior centrum semiovale and pons. Conversely, deviations in blood pressure below the optimal MAP were associated with reduced MD in the white matter of the cerebellum [32].

Based on the results of a limited number of experimental studies of cerebral blood flow in neonates and data from animal research, the following conclusions can be drawn. According to Manole et al. [33], when cerebral blood flow was assessed by MRI, cerebral blood flow was found to increase within 5 min after the primary episode of hypoxia-ischemia and to decrease after 10 min. In the study by Wang et al. [34], it was found that diastolic blood flow velocity increased significantly 3 h after the first episode of hypoxia-ischemia, as expressed by a lower resistance index (RI), which may indicate a decrease in the elasticity of cerebral blood vessels. Moreover, in a series of experiments investigating cerebral blood flow by Rosenberg et al. [35,36], it was found that cerebral blood flow increased 2 h after primary hypoxia-ischemia, but at the same time, the phase of reactive hyperemia was followed by a phase of hypoperfusion. The increase in oxygen delivery to brain cells is accompanied by a marked decrease in oxygen consumption, indicating mitochondrial dysfunction. In another study, a decrease in cerebral blood flow was also observed 20 min after reperfusion [37]. A study by Nakamura et al. found that an increase in cerebral blood flow within 6 h of primary hypoxia-ischemia indicates more severe brain cell damage [38]. However, according to the literature, hyperperfusion is not characteristic of all areas of the brain but is determined locally. Leffler et al., who studied hyperperfusion with radiolabeled microspheres, found that these phenomena occur in the cerebellum, diencephalon, midbrain, and medulla oblongata [37]. 

In relation to neonatal studies, a group of researchers led by Wu et al. [39] found that the increase in cardiac output during the rewarming phase after therapeutic hypothermia was due to an increase in heart rate and also an increase in peak systolic velocity measured in the MCA. In a study by Shaikh et al. [40], it was found that cerebral blood flow in neonates with hypoxic-ischemic encephalopathy increases by the 10th day of life and remains elevated until the age of one month. Cerebral hyperperfusion defined in full-term newborns with HIE correlates with adverse neurodevelopmental outcome [41]. Wintermark et al. examined cerebral blood flow in neonates who had suffered birth asphyxia. According to the authors, despite therapeutic hypothermia, hypoperfusion followed by hyperperfusion was observed during hypothermia in brain regions later identified as damaged [42]. The question of whether the increase in cerebral blood flow after hypoxia-ischemia is an adaptive physiological response of blood flow or a pathological response of cerebral vessels in patients with encephalopathy, including before, during, and after hypothermia sessions, currently remains unanswered [43].

### 2.2. Diagnosis and Treatment of NE

Understanding the pathophysiologic processes underlying primary vasoconstriction and secondary vasodilation leading to hyperperfusion will contribute to the early identification of neonates at high risk of severe brain injury [44]. Early diagnosis of cerebral hyperperfusion in neonates with HIE is necessary for the development of new treatment strategies for this patient population. The use of NIRS in the intensive care unit to measure cerebral perfusion can be started at any time. Cerebral perfusion depends on the blood pressure gradient (perfusion pressure) and cerebral vascular tone. By influencing these indicators, it is possible to change the indicators of cerebral perfusion. As a result of the effects of asphyxia on the fetus, the critical threshold of blood pressure required to maintain adequate cerebral perfusion may change. As cerebral perfusion decreases, central blood flow decreases during the first day of life and gradually increases over the next few days depending on the extent of the injury. These changes make the newborn brain more susceptible to ischemia with subsequent hyperperfusion. Therefore, the ability to monitor and optimize cerebral perfusion is critical to prevent ischemia and reperfusion injury [45]. Current treatment strategies for infants born with perinatal asphyxia have significantly improved survival rates and long-term adverse neurodevelopmental outcomes. However, full recovery of central nervous system function remains a challenge in some cases. Adequate assessment of pregnancy and birth history, appropriate resuscitation in the first minutes and hours of a newborn’s life, and adequate evaluation of criteria and indications for early therapeutic intervention all contribute to increased survival of infants born in the state of asphyxia. The only recommended and approved treatment for this type of patient is therapeutic hypothermia. It is believed that a decrease in body temperature reduces brain perfusion [46]. However, hypothermia alone cannot explain all aspects of decreased cerebral blood flow. Data from a study led by Wintermark et al. [47] suggest that encephalopathy itself also reduces cerebral blood flow. At the same time, determining how changes in cerebral blood flow depend on the severity of HIE and the effect on cerebral hemodynamics, including the relationship between brain perfusion and brain cell activity as measured by electroencephalogram (EEG), remains to be determined [48]. 

Examination of cerebral blood flow patterns is an important aspect of the evaluation of neonates in various critical states, especially those at risk for developing HIE. In order to diagnose intrauterine circulatory disorders, assess the possible development of neonatal encephalopathy, and evaluate the efficacy of the therapy administered, it is necessary to establish clear criteria. These criteria are based on a complex set of markers and indicators that correlate primarily with clinical outcomes [49] and include: assessment by Apgar score in the first 5 min of life [50], the need for cardiopulmonary resuscitation or intubation in the delivery room [51], a blood pH of 7.0 or less [52], pathologic neurologic symptoms [53], and assessment of brain electrical activity by amplitude-integrated EEG [54]. However, these markers for the risk of developing and forming neonatal encephalopathy are not definitive. For example, the average pH of arterial blood in a healthy fetus is about 7.35, whereas the average pH of umbilical artery blood at birth is about 7.25. According to the literature, a pH below 7.15 is a critical threshold that determines the risk of neonatal encephalopathy [55]. Adequate assessment of the neurological status of an asphyxiated neonate is also complex and depends on the specialist’s expertise. In addition, the performance of complex diagnostic procedures and interventions is limited by the severity of the condition and the ability to transport the infant. 

From a pathophysiological point of view, the main cause of a hypoxia and ischemia episode is an interruption in the oxygen supply to the cells of the brain due to inadequate blood supply. Therefore, the establishment and implementation of bedside diagnostic methods to assess the severity and risk of developing encephalopathy are of paramount importance. These methods include near-infrared spectroscopy (NIRS) and neurovisualization techniques based on ultrasound diagnostics.

## 3. Non-Invasive Assessment of Brain Perfusion

The diagnosis of criteria for the formation of neonatal encephalopathy is a complex of clinical, laboratory, and instrumental methods aimed at identifying and confirming the presence of persistent metabolic changes in systems and organs, limited in time by the presence of a strictly therapeutic window. Modern diagnostic methods and criteria for the risk of encephalopathy formation, such as assessment by the Apgar scale, determination of pH level, base deficit, assessment of neurological status, and evaluation of the electrical activity of the brain, both in combination and individually, may be biased or prone to errors at the pre-analytical and analytical stages and do not meet the requirements of an ideal biological marker for the formation of neonatal encephalopathy. MRI is one of the most accurate techniques for detecting abnormalities in HIE cases. To obtain high-quality imaging, however, sedation is often required, and extensive experience of an MRI specialist is needed for results interpretation. Also, MRI scanners are not as accessible in ordinary perinatal centers as ultrasound and NIRS machines. Therefore, we have focused our attention in this review on the latter two techniques. The search for the main literature sources was carried out in MEDLINE via the PubMed, Web of Science, and Scopus databases using the queries “neonatal encephalopathy”, “hypoxic-ischemic encephalopathy” “ultrasound” and “near-infrared spectroscopy”.

### 3.1. Ultrasound

Another noninvasive diagnostic method for assessing cerebral blood flow in newborns is ultrasound. In neonatal clinical practice, natural anatomical windows such as the anterior and posterior fontanelles, thin temporal bone, and mastoid process are used. Ultrasound is the method of choice in neonatology because it is widely available, does not contain ionizing radiation, and does not require sedation. Neurosonography has become an affordable and reliable technique for detecting focal intracerebral lesions, including intraventricular hemorrhage, infarcts, and hydrocephalus [56]. However, the detection of diffuse processes that may result from perinatal asphyxia, such as white matter edema, is proving more difficult and appears to be more dependent on physician expertise and experience. 

In neonate HIE, brain white matter echogenicity is increased compared to hypoechoic gray matter [57,58]. Several methods have been proposed for the diagnosis of neonatal encephalopathy. Simaeys et al., for example, used a computerized approach to minimize the subjective human factor in assessing the echogenicity of brain structures. The technique proposes a comparison between the echogenicity of intracerebral lesions and the relatively constant echogenicity of the choroid plexus [59]. Padilla et al. found that the calculation of relative echogenicity (the ratio of average pixel brightness measured in brain and bone regions at the same examination depth) can serve as a semiquantitative method for assessing different anatomic regions of the newborn brain [60]. 

Other effects of ultrasound are used to describe the properties of blood flow in the brain, such as the Doppler effect, in, in particular, spectral Doppler or pulsed wave ultrasonography. This means that the ultrasound probe emits short pulses of sound waves at a specific frequency and then listens for the echoes that bounce back from the moving blood cells. By analyzing the change in frequency of these echoes, the speed and direction of blood flow can be calculated and displayed as a spectral waveform. The collected Doppler signals are processed using Fourier analysis, a mathematical technique that decomposes complex waveforms into their individual frequency components. This analysis allows velocity information to be extracted from the Doppler spectrum. The resulting spectral waveform provides valuable information about blood flow patterns. The shape of the waveform reflects the characteristics of the flow, such as laminar, turbulent, or pulsatile. The height of the waveform is proportional to the speed of blood flow, with higher peaks indicating faster flow. Spectral broadening or narrowing can also provide information about changes in the flow pattern, such as stenosis or occlusion [61]. The use of Doppler ultrasound has become widespread in general clinical practice, including obstetrics and neonatology. In a study by Ehehalt et al., total cerebral blood flow was assessed by determining the volume of intravascular blood flow, calculated from time-averaged velocities, adjusted for the angle and cross-sectional area of the vessel in the extracranial internal carotid and vertebral arteries [62]. Information about cerebral blood flow, obtained by using this method, informs only about global intracerebral blood flow and cannot reflect regional changes. Other commonly used ultrasound Doppler instruments include color and power Doppler. While color Doppler is less sensitive to slow blood flow, power Doppler has higher sensitivity to blood flow in microvessels [63]. This characteristic makes power Doppler suitable for studying regional cerebral perfusion in neonates in cortical or deep structures such as the basal ganglia [64].

Blood flow velocity is commonly used in practice to describe the perfusion of an organ. The pulsatility index (PI), also known as the Gosling index, and the resistivity index (RI), also known as the Pourcelot index, are two parameters commonly used to evaluate the characteristics of blood flow in different vascular beds. These indices provide valuable information about the resistance and pulsatility of blood flow, which can be useful in the evaluation of various diseases and conditions affecting the vascular system. The pulsatility index is a quantitative measurement that reflects the pulsatile nature of blood flow in a given vessel. It is calculated by subtracting the peak systolic flow velocity from the end-diastolic flow velocity and dividing the result by the mean flow velocity. The formula for calculating the PI is as follows: PI = (Peak Systolic Velocity − End Diastolic Velocity)/Mean Velocity. PI values range from 0 to 1. Higher PI values indicate increased resistance and a more pulsatile flow pattern, usually associated with conditions such as arterial stiffness, vasoconstriction, or obstruction. Lower PI values indicate lower resistance and a less pulsatile flow pattern, which is often seen with conditions such as vasodilation or distal stenosis [65,66,67]. PI is commonly used to evaluate blood flow in various vascular beds, including the cerebral, renal, and fetal circulations. In the cerebral circulation, fluctuations in the PI value may indicate impaired autoregulation, altered perfusion pressure, or vascular abnormalities commonly seen in conditions such as stroke, intracranial hemorrhage, or cerebral vasospasm. The resistivity index is another parameter that provides information about the resistance of blood flow in a vessel. It is calculated by subtracting the end diastolic velocity from the peak systolic velocity and dividing the result by the peak systolic velocity. The formula for calculating the RI is as follows: RI = (Peak Systolic Velocity − End Diastolic Velocity)/Peak Systolic Velocity. Like the pulsatility index, the resistivity index ranges from 0 to 1. Higher RI values indicate increased resistance to blood flow, often seen in arterial narrowing, stenosis, or obstruction. Lower RI values indicate lower resistance and more efficient vascular perfusion [66,67]. PI and RI are related indicators of arterial blood flow that characterize the change in the velocity characteristics of the blood flow of an organ during a cardiac cycle [66]. On the basis of these indicators, it is possible to characterize flow pulsation, but at the same time, it is impossible to estimate resistance [66,68,69]. 

In neonates, on the basis of the indicators PI and RI measured in the anterior or middle cerebral artery, it is possible to make an objective functional assessment of hemodynamics and cerebral perfusion, as well as to predict the severity of the course and outcomes of HIE [70]. Studies have shown that RI changes in neonates who have experienced asphyxia [71]. An RI below 0.55 (measured in the anterior cerebral artery) has been found to correlate with unfavorable neurologic outcomes [72], especially when measured within the first 24–72 h after the first episode of hypoxic ischemia [73]. The results of a study by Gerner et al. also show that an RI value above 0.6 before the onset of therapeutic hypothermia is highly likely to identify neonates who will later develop severe neurologic problems at 20–32 months of age [74]. In addition, RI values above 0.6 after therapeutic hypothermia may be directly associated with worse functional outcomes in gross motor skills. This is consistent with a number of studies. An RI ≤ 0.55 has been associated with severe disability among children born under asphyxia [70,74,75,76,77,78,79,80,81,82,83,84], including neonates subjected to therapeutic hypothermia. According to Elstad et al., an RI ≤ 0.55 during therapeutic hypothermia (i.e., 48–72 h of life) has a sensitivity of 58% and a specificity of 83% for predicting outcome [76]. Skranes et al. suggested that a decrease in body temperature could lead to cerebral vasoconstriction and, consequently, an increase in RI values during a session of therapeutic hypothermia, whereas an RI ≤ 0.55 after rewarming has a sensitivity of 43% and a specificity of 100% for long-term predictions [77]. However, there are also reports in the literature suggesting that the RI obtained during a therapeutic hypothermia session is not predictive of outcome in neonates with HIE [77]. In addition, there is not much information on the relationship between the results of the RI and magnetic resonance imaging of the brain [85]. Assessment of blood flow characteristics of organs and systems during and after an episode of hypoxia and ischemia is one of the most important tasks in diagnosing the severity of brain injury.

### 3.2. Near-Infrared Spectroscopy

NIRS is a non-invasive diagnostic method based on the optical absorption of infrared light and its reflection from biological structures. Physically, NIRS is based on the interaction of near-infrared light with biological tissue. Near-infrared light with wavelengths between 650 and 1350 nm falls within the optical window where tissues have relatively low absorption and scattering properties allowing near-infrared light to penetrate biological tissues, including the brain, to a certain depth [86]. Biologically, NIRS takes advantage of the differential absorption of near-infrared light by oxygenated (oxyhemoglobin) and deoxyhemoglobin forms of hemoglobin. Oxyhemoglobin and deoxyhemoglobin have different absorption spectra in the near-infrared region. By measuring the intensity of transmitted or reflected light, NIRS can indirectly assess the relative concentrations of oxyhemoglobin and deoxyhemoglobin, providing information about tissue oxygen levels and blood supply. 

By monitoring changes in hemoglobin concentration and oxygen levels in real time, NIRS offers a non-invasive and continuous assessment of tissue blood supply, especially in critical care [87]. This technique is proving particularly useful in assessing cerebral blood flow in infants with conditions such as HIE. The apparatus’s sensors are attached to the scalp in the case of assessment of cerebral blood flow. The light penetrates the tissue and is scattered and absorbed before being detected by the sensor. Through advanced signal processing algorithms, the NIRS system can derive information such as regional cerebral oxygen saturation (rSO_2_), which provides an indication of the oxygen status and blood supply to the tissue being measured. One of the advantages of NIRS is its simplicity and portability, allowing it to be used directly at the patient’s bedside. Combining cerebral oxygenation monitoring with blood pressure measurements is an effective noninvasive method to continuously assess cerebral perfusion and oxygenation [88].

Continuous monitoring of cerebral perfusion is extremely important for patients with HIE. This is because these techniques make it possible to quickly detect decompensation [88]. At the same time, the application of this method is limited by the penetration depth of the light beam into the brain tissue (10–15 mm) [89]. Moreover, nowadays the data on outcomes after applying this method are just beginning to appear in the literature [90]. A recent meta-analysis by El-Atawi and colleagues showed that several NIRS parameters, such as regional cSpO_2_ and cerebral fractional tissue oxygen extraction, are significantly associated with adverse outcomes in the first 72 h after birth [91]. A prospective cohort study by Tewari et al. found that with brain rSO_2_ levels of 55–85% monitored by NIRS, this cohort had better neurodevelopmental outcomes at 12 months in infants with encephalopathy due to HIE and non-HIE etiologies [92]. Pereira and colleagues showed that after hypothermia, regional cerebral oxygen saturation values below 66.0% significantly predicted normal neurodevelopment, while values above 82% are associated with severe disability [93]. Brain perfusion studies using NIRS technology in term infants with severe HIE, who were not therapeutically hypothermic, have shown changes in cerebral blood flow and cerebral blood volume to predict poor outcome [72,94]. Among the trends in the development of NIRS monitoring, one can note the development of the predictive ability of disease outcome, the development of multimodular measurements [95] (for example, simultaneous assessment of cerebral oximetry and respiratory rate) [96], and the improvement of results analysis, including through the use of machine learning.

## 4. Conclusions

In this review, we examined the two most common non-invasive techniques used to assess the condition of encephalopathy in neonates born under asphyxia. Each is used both for diagnosis and for monitoring the patient’s condition, allowing one to evaluate the effectiveness of the therapy (for example, therapeutic hypothermia), and has its own predictive ability. Despite the fact that Doppler sonography currently has the greatest ability to predict the outcome, an array of NIRS data indicates the technique’s great prospects in this area. The combination of both bedside technologies, multimodality of measurements, the use of machine learning, and standardization of protocols will improve disease outcomes. Therefore, further research is needed to investigate the indices and characteristics of cerebral blood flow that are most appropriate for diagnosis and to understand the dynamics of changes in these features during therapeutic intervention. The expected results of such studies will allow a more accurate and rapid diagnosis of cerebral blood flow autoregulation abnormalities in this specific patient population and allow prediction of treatment outcomes.

## Data Availability

Data are available in a publicly accessible repository.

## References

[1] Lee A.C.C., Kozuki N., Blencowe H., Vos T., Bahalim A., Darmstadt G.L., Niermeyer S., Ellis M., Robertson N.J., Cousens S. (2013). Intrapartum-Related Neonatal Encephalopathy Incidence and Impairment at Regional and Global Levels for 2010 with Trends from 1990. Pediatr. Res..

[2] Aslam S., Strickland T., Molloy E.J. (2019). Neonatal Encephalopathy: Need for Recognition of Multiple Etiologies for Optimal Management. Front. Pediatr..

[3] Ferriero D.M. (2004). Neonatal Brain Injury. N. Engl. J. Med..

[4] Sandoval Karamian A.G., Mercimek-Andrews S., Mohammad K., Molloy E.J., Chang T., Chau V., Murray D.M., Wusthoff C.J. (2021). Neonatal Encephalopathy: Etiologies Other than Hypoxic-Ischemic Encephalopathy. Semin. Fetal Neonatal Med..

[5] Lorek A., Takei Y., Cady E.B., Wyatt J.S., Penrice J., Edwards A.D., Peebles D., Wylezinska M., Owen-Reece H., Kirkbride V. (1994). Delayed (“Secondary”) Cerebral Energy Failure after Acute Hypoxia-Ischemia in the Newborn Piglet: Continuous 48-Hour Studies by Phosphorus Magnetic Resonance Spectroscopy. Pediatr. Res..

[6] Chalak L., Ferriero D.M., Gressens P., Molloy E., Bearer C. (2019). A 20 Years Conundrum of Neonatal Encephalopathy and Hypoxic Ischemic Encephalopathy: Are We Closer to a Consensus Guideline?. Pediatr. Res..

[7] Dammann O., Ferriero D., Gressens P. (2011). Neonatal Encephalopathy or Hypoxic-Ischemic Encephalopathy? Appropriate Terminology Matters. Pediatr. Res..

[8] (2014). Executive Summary: Neonatal Encephalopathy and Neurologic Outcome, Second Edition. Report of the American College of Obstetricians and Gynecologists’ Task Force on Neonatal Encephalopathy. Obstet. Gynecol..

[9] Leon R.L., Mir I.N., Herrera C.L., Sharma K., Spong C.Y., Twickler D.M., Chalak L.F. (2022). Neuroplacentology in Congenital Heart Disease: Placental Connections to Neurodevelopmental Outcomes. Pediatr. Res..

[10] Roberts D.J., Polizzano C. (2021). Atlas of Placental Pathology.

[11] Mir I.N., Johnson-Welch S.F., Nelson D.B., Brown L.S., Rosenfeld C.R., Chalak L.F. (2015). Placental Pathology Is Associated with Severity of Neonatal Encephalopathy and Adverse Developmental Outcomes Following Hypothermia. Am. J. Obstet. Gynecol..

[12] Hirschel J., Barcos-Munoz F., Chalard F., Chiodini F., Epiney M., Fluss J., Rougemont A.-L. (2024). Perinatal Arterial Ischemic Stroke: How Informative Is the Placenta?. Virchows Arch..

[13] Pierrat V., Haouari N., Liska A., Thomas D., Subtil D., Truffert P., Groupe d’Etudes en Epidémiologie Périnatale (2005). Prevalence, Causes, and Outcome at 2 Years of Age of Newborn Encephalopathy: Population Based Study. Arch. Dis. Child. Fetal Neonatal Ed..

[14] Greco P., Nencini G., Piva I., Scioscia M., Volta C.A., Spadaro S., Neri M., Bonaccorsi G., Greco F., Cocco I. (2020). Pathophysiology of Hypoxic-Ischemic Encephalopathy: A Review of the Past and a View on the Future. Acta Neurol. Belg..

[15] Korf J.M., McCullough L.D., Caretti V. (2023). A Narrative Review on Treatment Strategies for Neonatal Hypoxic Ischemic Encephalopathy. Transl. Pediatr..

[16] Ravichandran L., Allen V.M., Allen A.C., Vincer M., Baskett T.F., Woolcott C.G. (2020). Incidence, Intrapartum Risk Factors, and Prognosis of Neonatal Hypoxic-Ischemic Encephalopathy Among Infants Born at 35 Weeks Gestation or More. J. Obstet. Gynaecol. Can..

[17] Hellwig L., Brada M., Held U., Hagmann C., Bode P., Frontzek K., Frey B., Brotschi B., Grass B. (2022). Association of Perinatal Sentinel Events, Placental Pathology and Cerebral MRI in Neonates with Hypoxic-Ischemic Encephalopathy Receiving Therapeutic Hypothermia. J. Perinatol..

[18] Sarnat H.B., Sarnat M.S. (1976). Neonatal Encephalopathy Following Fetal Distress. A Clinical and Electroencephalographic Study. Arch. Neurol..

[19] Tuiskula A., Metsäranta M., Toiviainen-Salo S., Vanhatalo S., Haataja L. (2022). Profile of Minor Neurological Findings after Perinatal Asphyxia. Acta Paediatr..

[20] Mathew J.L., Kaur N., Dsouza J.M. (2022). Therapeutic Hypothermia in Neonatal Hypoxic Encephalopathy: A Systematic Review and Meta-Analysis. J. Glob. Health.

[21] Bhagwani D.K. (2016). To Study the Correlation of Thompson Scoring in Predicting Early Neonatal Outcome in Post Asphyxiated Term Neonates. JCDR.

[22] Thayyil S., Pant S., Montaldo P., Shukla D., Oliveira V., Ivain P., Bassett P., Swamy R., Mendoza J., Moreno-Morales M. (2021). Hypothermia for Moderate or Severe Neonatal Encephalopathy in Low-Income and Middle-Income Countries (HE-LIX): A Randomised Controlled Trial in India, Sri Lanka, and Bangladesh. Lancet Glob. Health.

[23] Wladimiroff J.W., Tonge H.M., Stewart P.A. (1986). Doppler Ultrasound Assessment of Cerebral Blood Flow in the Human Fetus. BJOG.

[24] Cheung Y.F., Lam P.K.L., Yeung C.Y. (1994). Early Postnatal Cerebral Doppler Changes in Relation to Birth Weight. Early Hum. Dev..

[25] Noori S., Wlodaver A., Gottipati V., McCoy M., Schultz D., Escobedo M. (2012). Transitional Changes in Cardiac and Cerebral Hemodynamics in Term Neonates at Birth. J. Pediatr..

[26] Crockett S.L., Berger C.D., Shelton E.L., Reese J. (2019). Molecular and Mechanical Factors Contributing to Ductus Arteriosus Patency and Closure. Congenit. Heart Dis..

[27] De Vis J.B., Petersen E.T., De Vries L.S., Groenendaal F., Kersbergen K.J., Alderliesten T., Hendrikse J., Benders M.J.N.L. (2013). Regional Changes in Brain Perfusion during Brain Maturation Measured Non-Invasively with Arterial Spin Labeling MRI in Neonates. Eur. J. Radiol..

[28] Meek J.H., Elwell C.E., McCormick D.C., Edwards A.D., Townsend J.P., Stewart A.L., Wyatt J.S. (1999). Abnormal Cerebral Haemodynamics in Perinatally Asphyxiated Neonates Related to Outcome. Arch. Dis. Child. Fetal Neonatal Ed..

[29] Chen M.W., Lee J.K., Vezina G., Tekes A., Perin J., Li R., O’Kane A., McGowan M., Chang T., Parkinson C. (2022). The Utility of Cerebral Autoregulation Indices in Detecting Severe Brain Injury Varies by Cooling Treatment Phase in Neonates with Hypoxic-Ischemic Encephalopathy. Dev. Neurosci..

[30] Lee J.K., Poretti A., Perin J., Huisman T.A.G.M., Parkinson C., Chavez-Valdez R., O’Connor M., Reyes M., Armstrong J., Jennings J.M. (2017). Optimizing Cerebral Autoregulation May Decrease Neonatal Regional Hypoxic-Ischemic Brain Injury. Dev. Neurosci..

[31] Massaro A.N., Govindan R.B., Vezina G., Chang T., Andescavage N.N., Wang Y., Al-Shargabi T., Metzler M., Harris K., du Plessis A.J. (2015). Impaired Cerebral Autoregulation and Brain Injury in Newborns with Hypoxic-Ischemic Encephalopathy Treated with Hypothermia. J. Neurophysiol..

[32] Carrasco M., Perin J., Jennings J.M., Parkinson C., Gilmore M.M., Chavez-Valdez R., Massaro A.N., Koehler R.C., Northington F.J., Tekes A. (2018). Cerebral Autoregulation and Conventional and Diffusion Tensor Imaging Magnetic Resonance Imaging in Neonatal Hypoxic-Ischemic Encephalopathy. Pediatr. Neurol..

[33] Manole M.D., Foley L.M., Hitchens T.K., Kochanek P.M., Hickey R.W., Bayir H., Alexander H., Ho C., Clark R.S. (2009). Magnetic Resonance Imaging Assessment of Regional Cerebral Blood Flow after Asphyxial Cardiac Arrest in Immature Rats. J. Cereb. Blood Flow. Metab..

[34] Wang S.-D., Liang S.-Y., Liao X.-H., Deng X.-F., Chen Y.-Y., Liao C.-Y., Wang L., Tang S., Li Z.-X. (2015). Different Extent of Hypoxic-Ischemic Brain Damage in Newborn Rats: Histopathology, Hemodynamic, Virtual Touch Tissue Quantification and Neurobehavioral Observation. Int. J. Clin. Exp. Pathol..

[35] Rosenberg A.A. (1988). Regulation of Cerebral Blood Flow after Asphyxia in Neonatal Lambs. Stroke.

[36] Rosenberg A.A. (1986). Cerebral Blood Flow and O_2_ Metabolism after Asphyxia in Neonatal Lambs. Pediatr. Res..

[37] Leffler C.W., Busija D.W., Mirro R., Armstead W.M., Beasley D.G. (1989). Effects of Ischemia on Brain Blood Flow and Oxygen Consumption of Newborn Pigs. Am. J. Physiol. Heart Circ. Physiol..

[38] Nakamura M., Jinnai W., Hamano S., Nakamura S., Koyano K., Chiba Y., Kanenishi K., Yasuda S., Ueno M., Miki T. (2015). Cerebral Blood Volume Measurement Using Near-infrared Time-resolved Spectroscopy and Histopathological Evaluation after Hypoxic-ischemic Insult in Newborn Piglets. Int. J. Dev. Neurosci..

[39] Wu T.-W., Tamrazi B., Soleymani S., Seri I., Noori S. (2018). Hemodynamic Changes During Rewarming Phase of Whole-Body Hypothermia Therapy in Neonates with Hypoxic-Ischemic Encephalopathy. J. Pediatr..

[40] Shaikh H., Lechpammer M., Jensen F.E., Warfield S.K., Hansen A.H., Kosaras B., Shevell M., Wintermark P. (2015). Increased Brain Perfusion Persists over the First Month of Life in Term Asphyxiated Newborns Treated with Hypothermia: Does It Reflect Activated Angiogenesis?. Transl. Stroke Res..

[41] De Vis J.B., Hendrikse J., Petersen E.T., De Vries L.S., Van Bel F., Alderliesten T., Negro S., Groenendaal F., Benders M.J.N.L. (2015). Arterial Spin-Labelling Perfusion MRI and Outcome in Neonates with Hypoxic-Ischemic Encephalopathy. Eur. Radiol..

[42] Wintermark P., Hansen A., Gregas M.C., Soul J., Labrecque M., Robertson R.L., Warfield S.K. (2011). Brain Perfusion in Asphyxiated Newborns Treated with Therapeutic Hypothermia. Am. J. Neuroradiol..

[43] Perlman J.M. (2006). Summary Proceedings from the Neurology Group on Hypoxic-Ischemic Encephalopathy. Pediatrics.

[44] Kleuskens D.G., Gonçalves Costa F., Annink K.V., van den Hoogen A., Alderliesten T., Groenendaal F., Benders M.J.N., Dudink J. (2021). Pathophysiology of Cerebral Hyperperfusion in Term Neonates With Hypoxic-Ischemic Encephalopathy: A Systematic Review for Future Research. Front. Pediatr..

[45] Mitra S., Bale G., Meek J., Tachtsidis I., Robertson N.J. (2020). Cerebral Near Infrared Spectroscopy Monitoring in Term Infants With Hypoxic Ischemic Encephalopathy-A Systematic Review. Front. Neurol..

[46] Polderman K.H. (2008). Induced Hypothermia and Fever Control for Prevention and Treatment of Neurological Injuries. Lancet.

[47] Wintermark P., Hansen A., Soul J., Labrecque M., Robertson R.L., Warfield S.K. (2011). Early versus Late MRI in Asphyxiated Newborns Treated with Hypothermia. Arch. Dis. Child. Fetal Neonatal Ed..

[48] Lee S.J., Hatran D.P., Tomimatsu T., Peña J.P., McAuley G., Longo L.D. (2009). Fetal Cerebral Blood Flow, Electrocorticographic Activity, and Oxygenation: Responses to Acute Hypoxia. J. Physiol..

[49] Finer N.N., Robertson C.M., Richards R.T., Pinnell L.E., Peters K.L. (1981). Hypoxic-Ischemic Encephalopathy in Term Neonates: Perinatal Factors and Outcome. J. Pediatr..

[50] Levene M.I., Grindulis H., Sands C., Moore J.R. (1986). Comparison of Two Methods of Predicting Outcome in Perinatal Asphyxia. Lancet.

[51] Salhab W.A., Perlman J.M., Silver L., Sue Broyles R. (2004). Necrotizing Enterocolitis and Neurodevelopmental Outcome in Extremely Low Birth Weight Infants < 1000 g. J. Perinatol..

[52] Robertson N.J., Cowan F.M., Cox I.J., Edwards A.D. (2002). Brain Alkaline Intracellular pH after Neonatal Encephalopathy. Ann. Neurol..

[53] Horn E.-P., Bein B., Broch O., Iden T., Böhm R., Latz S.-K., Höcker J. (2016). Warming before and after Epidural Block before General Anaesthesia for Major Abdominal Surgery Prevents Perioperative Hypothermia: A Randomised Controlled Trial. Eur. J. Anaesthesiol..

[54] Weeke L.C., Boylan G.B., Pressler R.M., Hallberg B., Blennow M., Toet M.C., Groenendaal F., De Vries L.S. (2016). Role of EEG Background Activity, Seizure Burden and MRI in Predicting Neurodevelopmental Outcome in Full-Term Infants with Hypoxic-Ischaemic Encephalopathy in the Era of Therapeutic Hypothermia. Eur. J. Paediatr. Neurol..

[55] Nageotte M.P. (2015). Fetal Heart Rate Monitoring. Semin. Fetal Neonatal Med..

[56] Dudink J., Jeanne Steggerda S., Horsch S. (2020). State-of-the-Art Neonatal Cerebral Ultrasound: Technique and Reporting. Pediatr. Res..

[57] Childs A.M., Cornette L., Ramenghi L.A., Tanner S.F., Arthur R.J., Martinez D., Levene M.I. (2001). Magnetic Resonance and Cranial Ultrasound Characteristics of Periventricular White Matter Abnormalities in Newborn Infants. Clin. Radiol..

[58] Sie L.T., van der Knaap M.S., van Wezel-Meijler G., Taets van Amerongen A.H., Lafeber H.N., Valk J. (2000). Early MR Features of Hypoxic-Ischemic Brain Injury in Neonates with Periventricular Densities on Sonograms. Am. J. Neuroradiol..

[59] Simaeys B., Philips W., Lemahieu I., Govaert P. (2000). Quantitative Analysis of the Neonatal Brain by Ultrasound. Comput. Med. Imaging Graph..

[60] Padilla N.F., Enriquez G., Jansson T., Gratacos E., Hernandez-Andrade E. (2009). Quantitative Tissue Echogenicity of the Neonatal Brain Assessed by Ultrasound Imaging. Ultrasound Med. Biol..

[61] Pellett A.A., Kerut E.K. (2004). The Doppler Equation. Echocardiography.

[62] Ehehalt S., Kehrer M., Goelz R., Poets C., Schöning M. (2005). Cerebral Blood Flow Volume Measurements with Ultrasound: Interobserver Reproducibility in Preterm and Term Neonates. Ultrasound Med. Biol..

[63] Rubin J.M., Adler R.S., Fowlkes J.B., Spratt S., Pallister J.E., Chen J.F., Carson P.L. (1995). Fractional Moving Blood Volume: Estimation with Power Doppler US. Radiology.

[64] Heck S., Schindler T., Smyth J., Lui K., Meriki N., Welsh A. (2012). Evaluation of Neonatal Regional Cerebral Perfusion Using Power Doppler and the Index Fractional Moving Blood Volume. Neonatology.

[65] Bardelli M., Jensen G., Volkmann R., Aurell M. (1992). Non-Invasive Ultrasound Assessment of Renal Artery Stenosis by Means of the Gosling Pulsatility Index. J. Hypertens..

[66] Michel E., Zernikow B. (1998). Gosling’s Doppler Pulsatility Index Revisited. Ultrasound Med. Biol..

[67] Ciobanu A., Wright A., Syngelaki A., Wright D., Akolekar R., Nicolaides K.H. (2019). Fetal Medicine Foundation Reference Ranges for Umbilical Artery and Middle Cerebral Artery Pulsatility Index and Cerebroplacental Ratio. Ultrasound Obstet. Gynecol..

[68] Gosling R.G., Lo P.T.S., Taylor M.G. (1991). Interpretation of Pulsatility Index in Feeder Arteries to Low-impedance Vascular Beds. Ultrasound Obstet. Gynecol..

[69] Gómez O., Figueras F., Fernández S., Bennasar M., Martínez J.M., Puerto B., Gratacós E. (2008). Reference Ranges for Uterine Artery Mean Pulsatility Index at 11–41 Weeks of Gestation. Ultrasound Obstet. Gynecol..

[70] Liu J., Cao H.-Y., Huang X.-H., Wang Q. (2007). The Pattern and Early Diagnostic Value of Doppler Ultrasound for Neonatal Hypoxic-Ischemic Encephalopathy. J. Trop. Pediatr..

[71] Chao C.P., Zaleski C.G., Patton A.C. (2006). Neonatal Hypoxic-Ischemic Encephalopathy: Multimodality Imaging Findings. Radiographics.

[72] Archer L.N., Levene M., Evans D. (1986). Cerebral Artery Doppler Ultrasonography for Prediction of Outcome after Perinatal Asphyxia. Lancet.

[73] Ilves P., Lintrop M., Talvik I., Muug K., Maipuu L., Metsvaht T. (2009). Low Cerebral Blood Flow Velocity and Head Circumference in Infants with Severe Hypoxic Ischemic Encephalopathy and Poor Outcome. Acta Paediatr..

[74] Gerner G.J., Burton V.J., Poretti A., Bosemani T., Cristofalo E., Tekes A., Seyfert D., Parkinson C., Leppert M., Allen M. (2016). Transfontanellar Duplex Brain Ultrasonography Resistive Indices as a Prognostic Tool in Neonatal Hypoxic-Ischemic Encephalopathy before and after Treatment with Therapeutic Hypothermia. J. Perinatol..

[75] Jongeling B.R., Badawi N., Kurinczuk J.J., Thonell S., Watson L., Dixon G., Stanley F.J. (2002). Cranial Ultrasound as a Predictor of Outcome in Term Newborn Encephalopathy. Pediatr. Neurol..

[76] Elstad M., Whitelaw A., Thoresen M. (2011). Cerebral Resistance Index Is Less Predictive in Hypothermic Encephalopathic Newborns. Acta Paediatr..

[77] Skranes J.H., Elstad M., Thoresen M., Cowan F.M., Stiris T., Fugelseth D. (2014). Hypothermia Makes Cerebral Resistance Index a Poor Prognostic Tool in Encephalopathic Newborns. Neonatology.

[78] Levene M.I., Evans D.H., Forde A., Archer L.N. (1987). Value of Intracranial Pressure Monitoring of Asphyxiated Newborn Infants. Dev. Med. Child. Neurol..

[79] Eken P., Toet M.C., Groenendaal F., de Vries L.S. (1995). Predictive Value of Early Neuroimaging, Pulsed Doppler and Neurophysiology in Full Term Infants with Hypoxic-Ischaemic Encephalopathy. Arch. Dis. Child. Fetal Neonatal Ed..

[80] Low J.A. (1995). Cerebral Perfusion, Metabolism, and Outcome. Curr. Opin. Pediatr..

[81] Stark J.E., Seibert J.J. (1994). Cerebral Artery Doppler Ultrasonography for Prediction of Outcome after Perinatal Asphyxia. J. Ultrasound Med..

[82] Liao H.T., Hung K.L. (1997). Anterior Cerebral Artery Doppler Ultrasonography for Prediction of Outcome after Perinatal Asphyxia. Zhonghua Min Guo Xiao Er Ke Yi Xue Hui Za Zhi.

[83] Ilves P., Lintrop M., Talvik I., Muug K., Maipuu L. (2009). Changes in Cerebral and Visceral Blood Flow Velocities in Asphyxiated Term Neonates with Hypoxic-Ischemic Encephalopathy. J. Ultrasound Med..

[84] Pinto P.S., Tekes A., Singhi S., Northington F.J., Parkinson C., Huisman T.a.G.M. (2012). White-Gray Matter Echogenicity Ratio and Resistive Index: Sonographic Bedside Markers of Cerebral Hypoxic-Ischemic Injury/Edema?. J. Perinatol..

[85] Epelman M., Daneman A., Kellenberger C.J., Aziz A., Konen O., Moineddin R., Whyte H., Blaser S. (2010). Neonatal Encephalopathy: A Prospective Comparison of Head US and MRI. Pediatr. Radiol..

[86] Smith A.M., Mancini M.C., Nie S. (2009). Bioimaging: Second Window for in Vivo Imaging. Nat. Nanotechnol..

[87] Garvey A.A., Dempsey E.M. (2018). Applications of near Infrared Spectroscopy in the Neonate. Curr. Opin. Pediatr..

[88] Claessens N.H.P., Jansen N.J.G., Breur J.M.P.J., Algra S.O., Stegeman R., Alderliesten T., Van Loon K., De Vries L.S., Haas F., Benders M.J.N.L. (2019). Postoperative Cerebral Oxygenation Was Not Associated with New Brain Injury in Infants with Congenital Heart Disease. J. Thorac. Cardiovasc. Surg..

[89] Patil A.V., Safaie J., Moghaddam H.A., Wallois F., Grebe R. (2011). Experimental Investigation of NIRS Spatial Sensitivity. Biomed. Opt. Express.

[90] Lin N., Flibotte J., Licht D.J. (2018). Neuromonitoring in the Neonatal ECMO Patient. Semin. Perinatol..

[91] El-Atawi K.M., Osman M.F., Hassan M., Siwji Z.A., Hassan A.A., Abed M.Y., Elsayed Y. (2023). Predictive Utility of Near-Infrared Spectroscopy for the Outcomes of Hypoxic-Ischemic Encephalopathy: A Systematic Review and Meta-Analysis. Cureus.

[92] Tewari V.V., Kumar A., Kurup A., Daryani H., Saxena A. (2022). Impact of Cerebral Oxygen Saturation Monitoring on Short-Term Neurodevelopmental Outcomes in Neonates with Encephalopathy—A Prospective Cohort Study. Curr. Pediatr. Rev..

[93] Oliveira Pereira C., Dias A., Nunes Vicente I., Pinto J.T., Marques C., Dinis A., Pinto C., Carvalho L. (2021). Prognostic value of near-infrared spectroscopy in hypoxic-ischaemic encephalopathy. An. Pediatría.

[94] Ancora G., Maranella E., Grandi S., Sbravati F., Coccolini E., Savini S., Faldella G. (2013). Early Predictors of Short Term Neurodevelopmental Outcome in Asphyxiated Cooled Infants. A Combined Brain Amplitude Integrated Electroencephalography and near Infrared Spectroscopy Study. Brain Dev..

[95] Variane G.F.T., Pietrobom R.F.R., Noh C.Y., Van Meurs K.P., Chock V.Y. (2023). Newer Indications for Neuromonitoring in Critically Ill Neonates. Front. Pediatr..

[96] Hakimi N., Shahbakhti M., Horschig J.M., Alderliesten T., Van Bel F., Colier W.N.J.M., Dudink J. (2023). Respiratory Rate Extraction from Neonatal Near-Infrared Spectroscopy Signals. Sensors.

